# The variants question: What is the problem?

**DOI:** 10.1002/jmv.27196

**Published:** 2021-07-28

**Authors:** Davide Zella, Marta Giovanetti, Francesca Benedetti, Francesco Unali, Silvia Spoto, Michele Guarino, Silvia Angeletti, Massimo Ciccozzi

**Affiliations:** ^1^ Department of Biochemistry and Molecular Biology, Institute of Human Virology and Global Virus Network Center University of Maryland School of Medicine Baltimore Maryland USA; ^2^ Laboratório de Flavivírus, Instituto Oswaldo Cruz, Fundação Oswaldo Cruz Rio de Janeiro Brazil; ^3^ Laboratório de Genética Celular e Molecular, ICB, Universidade Federal de Minas Gerais Belo Horizonte Minas Gerais Brazil; ^4^ Area Comunicazione e Brand Management, University Campus Bio‐Medico of Rome Rome Italy; ^5^ Department of Diagnostic and Therapeutic Medicine University Campus Bio‐Medico of Rome Rome Italy; ^6^ Department of Gastrointestinal Diseases Campus Bio‐Medico University Rome Italy; ^7^ Unit of Clinical Laboratory Science, University Campus Bio‐Medico of Rome Rome Italy; ^8^ Medical Statistic and Molecular Epidemiology Unit, University of Biomedical Campus Rome Italy

**Keywords:** intervention strategies, pandemic, SARS‐CoV‐2, vaccine, VOCs, VOIs

## Abstract

The severe acute respiratory syndrome coronavirus 2 (SARS‐CoV‐2) originated in Wuhan, China in early December 2019 has rapidly widespread worldwide. Over the course of the pandemic, due to the advance of whole‐genome sequencing technologies, an unprecedented number of genomes have been generated, providing both invaluable insights into the ongoing evolution and epidemiology of the virus and allowing the identification of hundreds of circulating genetic variants during the pandemic. In recent months variants of SARS‐CoV‐2 that have an increased number of mutations on the Spike protein have brought concern all over the world. These have been called “variants of concerns” (VOCs), and/or “variants of interests” (VOIs) as it has been suggested that their genome mutations might impact transmission, immune control, and virulence. Tracking the spread of emerging SARS‐CoV‐2 variants is crucial to inform public health efforts and control the ongoing pandemic. In this review, a concise characterization of the SARS‐CoV‐2 mutational patterns of the main VOCs and VOIs circulating and cocirculating worldwide has been presented to determine the magnitude of the SARS‐CoV‐2 threat to better understand the virus genetic diversity and its potential impact on vaccination strategy.

## Highlights

- Over the course of the pandemic an unprecedented number of SARS‐CoV‐2 genomes have been generated.
- Genomic monitoring allowed the identification of “variants of concerns” (VOCs), and/or “variants of interests” (VOIs) wordlwide.
- Tracking the spread of emerging SARS‐CoV‐2 variants is crucial to control the ongoing pandemic.

## 1 INTRODUCTION

“Whether it is nobler to the soul to endure the outrages, stones, and darts of unfair fortune, or take up arms against a sea of trouble and fight to disperse them” quoting a historic sentence from Hamlet in a famous drama by William Shakespeare (Act III, Scene I) written in 1600–1602, where Hamlet's many doubts led him to claim, “the obstacles (in that sleep of death … from the development of this mortal life…) must induce us to reflect.”

We are living today an important phase of severe acute respiratory syndrome coronavirus 2 (SARS‐CoV‐2) pandemic where we identify different mutations and variants of the virus circulating all over the world.

Zoonoses are infectious diseases transmitted from animals to humans that can evolve to become efficiently transmissible human‐to‐human infections. The current pandemic caused by the SARS‐CoV‐2 virus is believed to have originated from a wildlife food market in China's Wuhan city towards the end of 2019.^1^, ^2^ Current evidence points to its origin from a bat‐borne virus and the global pandemic represents the first time that the virus has been transmitted into humans.^3^, ^4^

Generally, the rates of nucleotide substitution of RNA viruses are fast, and this rapid evolution is mainly shaped by natural selection. This high error rate and the consequent rapidly evolving virus populations,^5^ which could lead to the accumulation of amino acid mutations, might affect the transmissibility of the virus, its cell tropism, and pathogenicity. It would unfortunately also present daunting challenges for the design of effective vaccines and diagnostic assays. Fortunately, however, until now the observed diversity among SARS‐CoV‐2 sequences has been low.

Coronaviruses such as SARS‐CoV‐2 are relatively stable thanks to a proofreading mechanism that operates during replication. Many genomic studies have nevertheless revealed changes in their genomes, including mutations and deletions. As the terms mutation, variant, and strain are often used interchangeably in describing the epidemiology of SARS‐CoV‐2, the distinctions appear to be crucial. The genetic material of SARS‐CoV‐2 is RNA. To replicate, and therefore establish the infection, the virus must hijack the host cell and use the cell's machinery to duplicate itself. Errors often occur during this entire process which is the RNA replication. This results in viruses that are similar but not exact copies of the original one. Those errors are called mutations, and viruses with these mutations are called variants. Variants could differ by a single or many mutations. A variant is referred to as a strain when it starts to present distinct physical properties. Such differences could involve a variant binding to a different cell receptor, or replicating more quickly, or transmitting more efficiently, and enhancement in its virulence. Essentially, all strains are variants, but not all variants are strains.

The first SARS‐CoV‐2 variant with a D614G substitution in the spike protein emerged early in the pandemic, between January and February 2020.^6^, ^7^, ^8^, ^9^, ^10^ Over a period of several months, the D614G mutation replaced the initial SARS‐CoV‐2 strain identified in Wuhan, China, and by June 2020 became the dominant form of the virus circulating globally. Studies highlighted the role of this variant which appears to confer a fitness advantage to the virus possibly associated with the improvement of replication and/or transmission in humans.^8^, ^11^, ^12^

**Figure 1:**
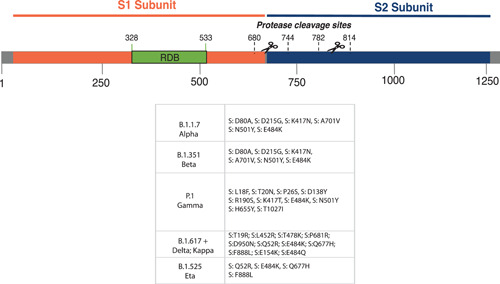
Schematic representation of S1 and S2 subunits of SARS‐CoV‐2 with the main mutations indicated for each VOC and VOI. The “+” symbol indicates the B.1.617.1 and B.1.617.2 variants. SARS‐CoV‐2, severe acute respiratory syndrome coronavirus 2; VOC, variants of concerns; VOI, variants of interests

Currently, four variants (B.1.1.7 also known as 20I/501Y.V1 or VOC 202012/01 or Alpha variant, B.1.351 also known as 20H/501Y.V2 or VOC 202012/02 or Beta variant, P.1 also known as 20J/501Y.V3 or Gamma variant and the B.1.617.2 also know as Delta variant) carrying several mutations in the receptor‐binding domain (RBD) of the spike (S) protein, raise concerns about their potential to shift the dynamics and public health impact of the pandemic.^13^, ^14^, ^15^, ^16^ They appear potentially associated with (i) increased transmissibility, (ii) propensity for re‐infection, (iii) escape from neutralizing antibodies, and (iv) increased affinity for the human angiotensin‐converting enzyme 2 (ACE2) receptor^16^, ^17^ (Figure 1 and Table 1).

**Table 1:** List of the main variants of concerns (VOC) and variants of interests (VOI) with the new WHO nomenclature, the old nomenclature based on lineages, the defining SNPs, the earliest documented samples dates, the location of the first detection, and the classification status

Variants	WHO nomenclature	Defining SNPs	Earliest sample date	First detected	Status
B.1.1.7	Alpha	aa:orf1ab:T1001I; aa:orf1ab:A1708D;	Sep 2020	UK	VOC
		aa:orf1ab:I2230T			
		del:11288:9; del:21765:6; del:21991:3			
		aa:S:N501Y; aa:S:A570D; aa:S:P681H; aa:S:T716I; aa:S:S982A; aa:S:D1118H; aa:Orf8:Q27*; aa:Orf8:R52I; aa:Orf8:Y73C			
		aa:N:D3L; aa:N:S235F			
B.1.351	Beta	aa:E:P71L; aa:N:T205I; aa:orf1a:K1655N; aa:S:D80A	May 2020	South Africa	VOC
		aa:S:D215G; aa:S:K417N; aa:S:A701V; aa:S:N501Y; aa:S:E484K			
P.1	Gamma	aa:orf1ab:S1188L	May 2020	Brazil	VOC
		aa:orf1ab:K1795Q; del:11288:9			
		aa:S:L18F; aa:S:T20N; aa:S:P26S; aa:S:D138Y; aa:S:R190S			
		aa:S:K417T; aa:S:E484K; aa:S:N501Y; aa:S:H655Y; aa:S:T1027I			
		aa:orf3a:G174C; aa:orf8:E92K; aa:N:P80R			
B.1.617.2	Delta	aa:S:T19R; aa:S:L452R; aa:S:T478K; aa:S:P681R; aa:S:D950N	Oct 2020	India	VOC
		aa:ORF3a:S26L; aa:M:I82T; aa:ORF7a:V82A; aa:ORF7a:T120I			
		aa:N:D63G; aa:N:R203M; aa:N:D377Y			
B.1.525	Eta	aa:orf1ab:L4715F; aa:S:Q52R; aa:S:E484K; aa:S:Q677H	Dec 2020	Nigeria	VOI
		aa:S:F888L; aa:E:L21F; aa:E:I82T; del:11288:9; del:21765:6			
		del:28278:3			
B.1.617.1	Kappa	aa:orf1ab: T1567I; aa:orf1ab: T3646A; aa:orf1ab: M5753I	Oct 2020	India	VOI
		aa:orf1ab: K6711R; aa:S: E154K; aa:S: L452R; aa:S: E484Q			
		aa:S: P681R; aa:orf3a: S26L; aa:orf7a: V82A; aa:N: R203M			

Abbreviation: SNPs, single nucleotide polymorphisms.

The Alpha variant contains 23 nucleotide substitutions and it is not phylogenetically related to the SARS‐CoV‐2 virus circulating in the UK at the time the variant was detected. Recently epidemiological modeling, phylogenetic and clinical findings suggest that the Alpha variant has increased transmissibility due to a mutation called N501Y, which allows SARS‐CoV‐2 to bind more readily to the human receptor ACE2, the entry point for SARS‐CoV‐2 to a wide range of human cells. However, preliminary analyses also indicate that there is no change in disease severity or occurrence of reinfection. Another mutation in this variant of concern (VOC), the deletion at position 69/70del, was found to affect the performance of some diagnostic polymerase chain reaction (PCR) assays with an S gene target. However, most PCR assays in use worldwide use multiple targets, and therefore the impact of the variant on diagnostics is not anticipated to be significant. Laboratory evaluation has also demonstrated no significant impact on the performance of antigen‐based lateral flow devices (Figure 1 and Table 1).

The Beta variant, also called the 501Y.V2 variant (because of the presence of the N501Y mutation), was first notified on December 18, 2020 by national authorities in South Africa. This variant is defined by eight mutations in the spike protein, including three substitutions (K417N, E484K, and N501Y) at residues in its RBD. In a few weeks, this variant largely replaced other SARS‐CoV‐2 viruses circulating in the Eastern Cape, Western Cape, and KwaZulu‐Natal provinces. Recent studies suggest this variant is associated with a higher viral load, which may suggest the potential for increased transmissibility, and or virulence, allowing differential clinical outcomes. Further investigations are needed to understand the impact on transmission, clinical severity of infection, laboratory diagnostics, therapeutics, vaccines, or public health preventive measures (Figure 1 and Table 1).

The Gamma variant, first identified in January 2021 in travelers from the Amazonas state (North of Brazil) who arrived in Japan,^15^ harbors a constellation of 17 unique mutations, including three in the RBD of the spike protein (K417T, E484K, and N501Y). It thus immediately raised concerns to public health authorities over the risk of its unknown potential of faster spreading and/or worsening of coronavirus disease 2019 (COVID‐19) clinical outcomes (Figure 1 and Table 1).

The Kappa variant, first identified in India in October 2020, has raised global concern, after being reported in many countries worldwide. This variant harbors a constellation of 12 unique mutations, including three in the RBD of the spike protein (T19R, L452R, T478K, P681R, and D950N) (Figure 1 and Table 1).

Since the identification of these VOCs, the combination between the unprecedented number of cases and more than 1.4 million genomes has allowed the identification of many other circulating genetic variants during the pandemic, containing lineage‐specific mutations including single point mutations and/or genetic deletions,^5^, ^18^, ^19^, ^20^, ^21^ most of them described as VOIs, including the B.1.525 (Eta variant) and the B.1.617.1 (Kappa variant) (Figure 1 and Table 1).

The Kappa variant, first identified in India in October 2020, has raised global concern, after being reported in many countries worldwide.^22^ This variant contains two key mutations to the outer spike portion of the virus, referred to as E484Q and L452R, which appear to have never been reported together. The identification of this double mutation in key areas of the virus's spike protein may allow the virus to escape the immune system (Figure 1 and Table 1).

The Eta variant has been described as a VOI for the first time in mid‐December 2020 in Nigeria and later then in many other countries. This variant presents a notable group of missense mutations believed to be of particular importance due to their potential for increased transmissibility, virulence, and reduced effectiveness of vaccines.^16^ Also known as 20A/S:484K, the Eta variant harbors some genetic signatures related to diverse putative effects on viral fitness, which are shared by other VOCs, highlighting the potential impact of dissemination in countries where it has not yet been detected: (i) Q677H—described as modulating transmissibility; (ii) Δ144—associated with immune escape; (iii) Δ106‐108—detected already in Alpha, Beta, and Gamma variants; (iv) E484K—present in Beta, Gamma, and Zeta variants; (v) N439K—found in Y453F, B.1.141, and B.1.258 variants; (vi) two deletions ΔH69/ΔV70 detected in Alpha variant^23^ (Figure 1 and Table 1).

S1 spike is the major SARS‐CoV‐2 protein allowing virus entry into human cells expressing ACE2 receptor through a defined RBD.^24^ It has been shown that antibodies generated against the spike protein can block the entry of SARS‐CoV‐2 into host cells, thus preventing infection.^25^ For this reason, many efforts to generate safe and efficacious COVID‐19 vaccines have been focused on targeting the spike protein. Changes in its sequence and structure are thus particularly relevant because they may hamper the ability of antibodies to bind and block the entry of the virus into the host cells.

The S1 mutation is in a position (AA 614; SD2 region) close to the S1 junction with the S2 component of the Spike protein. The mutation appears to diminish protein stability and change the conformation of the spike glycoprotein, thus enhancing receptor‐binding capacity, reducing the shedding, and increasing infectivity.^12^

Among other mutated proteins potentially important we also list Nsp1, which is also known as the leader protein and is central in the inhibition of the antiviral innate immune response. In particular, in cells infected by SARS‐CoV‐2, it has been shown to play a central role in reducing the expression of IFN‐α, and thus it is considered one of the most important determinants of viral pathogenicity.^26^ By analyzing genomes alignments from all over the world, our group identified a deletion of nine nucleotides in positions 686–694 (AA position 241–243) of the Nsp1 protein.^5^ Even if this deletion was present in only a few genomes from different geographic areas, structural prediction modeling suggested an alteration in the C‐terminal tail structure. It is possible that viruses harboring this deletion are likely to be less pathogenic than commonly observed viral strains, though more studies are needed to confirm this theory. To further support this concept, we note that two common endemic human coronaviruses, HCoV‐OC43^16^ and HCoV‐299E,^27^ have extensive deletions in the C‐terminal region of Nsp1.

To provide further information about how quickly the virus could potentially increase its genetic variability, we analyzed the Open Reading Frame 1ab gene of SARS‐CoV‐2 to look for the presence of mutations that could have been caused by selective pressure on the virus and that could influence its ability to infect the host.^28^ We found some potential sites under positive selective pressure and some other stabilizing mutations in the nsp2 protein that probably could explain why this virus is more contagious than SARS‐CoV.

Recently our group investigated SARS‐CoV‐2 transmission dynamics in Italy, one of the countries hit first and hardest by the pandemic, by coupling phylodynamic analysis of viral genetic and epidemiological data. Our study revealed that multiple SARS‐CoV‐2 lineages, largely linked to outside introductions, cocirculated during early epidemic spread, initially characterized by large transmission clusters concomitant with a high number of infections. Subsequent implementation of three‐phase nationwide lockdown measures greatly reduced infection and hospitalization cases during the summer, yet we demonstrate the persistence of viral spread among few, much smaller clusters acting as “hidden reservoirs.” Mathematical modeling shows that increased mobility among residents eventually catalyzed the coalescence of such clusters, thus increasing the number of infections and igniting a new epidemic wave. Our results suggest that timely identification and characterization of the epidemic reservoirs' number, size, and distribution may help to increase the effectiveness of containment measures and provide guidance for future vaccine deployment strategies.^29^ The probable failure of molecular surveillance systems to monitor the spread of different virus lineages in many countries has given rise to a large amount and spread of different genetic variants of SARS‐CoV‐2. Fortunately, there has been relatively limited evidence of virus mutations having a significant functional effect on the virus. Of importance is a data sharing and online platform to track in real‐time the lineages spread, giving new information to researchers all over the world.^30^ It is of importance to maintain the mutations landscape of SARS‐CoV‐2 under control globally to better understand the effect on the infectivity and antigenicity of the variants. Fortunately, most mutations are of little importance from the evolutionary point of view, and no consequence we have seen, but sometimes, the virus can acquire a mutation that gives it an advantage over other strains. In this case, the virus evolution can lead to a consequence, such as greater infectivity and scarce efficacy in antibody response. The Spike mutations can potentially facilitate better affinity or binding and enable easier entry into the host cell, as in the case of the D614G mutation. The RBD in the spike protein is the most variable part of the coronavirus genome.^2^ Mutations can putatively also render the virus resistant to neutralization by host antibodies.

A number of vaccines have been approved by several regulatory agencies worldwide, and at the time of writing the ones with the most scientific information available and most broadly used are BNT162b2 (Pfizer BioNTech),^31^ mRNA‐1273 (Moderna),^32^ Ad26.COV2.S (Jannsen),^33^ AZD1222 (AstraZeneca),^34^ and Sputnik‐V (Gam‐COVID‐Vac).^35^ The efficacy seems to be pretty high for all the vaccines, with 70%–95% protection against mild to severe COVID‐19 symptoms and almost total protection against death. However, several strains of SARS‐CoV‐2 carrying mutations in the spike protein were recently identified, such as the Alpha,^36^, ^37^ the Gamma,^14,15^ and the Beta variant.^14^ Mutations, forming variants that maintain virulence and viral fitness need to be identified and monitored to inform the future of COVID‐19 vaccines and therapeutics.

There are a number of preliminary studies aimed at evaluating the efficacy of each vaccine against these variants. Preliminary data seem to indicate that some vaccines are still effective, though they may need to be updated periodically.^38^, ^39^, ^40^, ^41^ The hope that three vaccines recently approved by the FDA for emergency use could determine the end of the SARS‐CoV‐2 pandemic has been supported by the evidence that these vaccines showed an efficacy superior to 85%. This hope has been dampened by the identification of viral variants with mutated spike protein, which in all vaccines is the viral antigen used for active immunization, thus worrying the public opinion with the suspicion that these variants could lower the vaccine's efficacy. So far evidence came from in vitro observations showing a 10‐fold decrease of neutralization antibody in presence of the viral variants, which in turn raised doubts and questions about vaccine efficacy against mutated SARS‐CoV‐2. However, more data are needed to validate these observations, and studies are also ongoing by challenging vaccinated individuals with active viruses and monitoring the infection development.^42^

Vaccine ineffectiveness is so worrying because it represents the real chance to fight the SARS‐CoV‐2 pandemic. The emergence of viral variants threatening this unique opportunity is the consequence of scientific evidence for mutations inducing enhanced virulence, re‐infection, and resistance to monoclonal or polyclonal antibodies therapy. In the case of antibody resistance, the T cell response should be evaluated to understand if the whole immune response could be affected or maintained. Contextually, vaccine production companies are acting by remodeling the first vaccine in the light of the new variants' appearance, even though this could be a continuous evolution phenomenon driven by the selective pressure exerted by the immune response and target viral therapy to which the virus is ever more subjected.^43^

The real meaning of SARS‐CoV‐2 variants and their impact on the vaccination campaign is the most important factor to consider. The viral variants affecting the spike protein represent the most important threat to vaccine efficacy, because of the potential decrease in antibody efficacy. This possibility seems improbable until population vaccine coverage will not be sufficient. Moreover, it is important to evaluate the size of neutralization activity change in case of infection sustained by viral strains carrying spike protein mutations. The neutralizing antibody titers routinely determined should be considered. In addition, the occurrence of a viral mutation increasing a determined characteristic of the virus could on the other side affect and decrease another property of the viral strains. On this basis, what could be useful now for the virus should not be in the next future making it less fit and more susceptible to the host immune response. For this reason, it is very important to consider SARS‐CoV‐2 evolution dynamics and host immune response by long‐term analysis.^44^

## 2 CONCLUSIONS

The COVID‐19 pandemic has stressed our health care systems in unprecedented ways and underlined once more the important role of the studies regarding viral molecular evolution to identify single points mutations and recombination events to assess the following possibilities: that two different SARS‐CoV‐2 strains may coinfect the same cell; and/or a SARS‐CoV‐2 strain might have acquired new traits like virulence and drug susceptibility directly from other strains^21^, ^24^; and/or the adaptability of SARS‐CoV‐2 to human immune system might be significantly strengthened through genetic recombination. For these reasons, the accuracy of diagnosis based on serologic and molecular biology assays might be compromised by this genetic variability,^25^ and also the transmission tracking based on the phylogenetic tree could be misleading since the topology of mutation route is a network rather than a tree.^45^, ^46^, ^47^

Additionally, it is necessary to understand the phenotypic impact of the SARS‐CoV‐2 mutations generating variants. Through infectivity assays and neutralization assays, it is possible to understand the effects of emerging mutations on ACE2 binding and NAb binding.

It is unlikely that the early viral mutations observed in Europe and then in the United States once the virus emerged from China radically influenced its fitness. Moreover, their contribution to lethality is difficult to determine, since at the beginning of a pandemic event, the virus is likely to be very aggressive and its sequences would tend to be more homogenous. On the other hand, the early mutations we observed in the viral polymerase gene could have affected its processivity and fidelity, further increasing its mutation rate and the generation of viral clades progressively more heterogeneous. The emergence of subsequent specific patterns of mutations, concomitant with the decline in case fatality rate, likely follows the principle of homoplasy and suggests a converged evolution due to the accumulation of mutations over time. This would, in turn, lead to the rapidly progressive and convergent adaptation of the virus to the human host. Additional confirmation and the biological significance of such mutations need to be determined. Nonetheless, it is tempting to speculate that they may contribute to the loss of virulence of SARS‐CoV‐2.

What we have known all along is the ability of the virus to mutate, without warning and without changing its characteristics in the interaction with humans. Mutations are significant only in a few cases and become interesting only when they are part of its strategy of adaptation and survival.

In parallel to the scientific discussion over time in the society of real‐time information, faced with a “minute‐by‐minute” story of the main global event (a planetary emergency in a hyper‐connected world), the traditional approach of the media has proved to be quite ineffective in explaining this phenomenon. Unfortunately, very often, the journalistic‐chronicle angle has prevailed over the scientific‐dissemination point of view.

The discovery of variants was indeed considered as a sudden event and presented with astonishment when it was an unpredictable (but expected) event that only deserved to be accurately explained.

A first observation: when did we start talking about variants? We started when concerns emerged about the so‐called “English variant” (Alpha variant). Until that moment topics of mutations and variants had always remained in the background. Mutations were just a possibility that the virus was not yet expressing one important way, not least because for several months many insignificant variants had been observed. For months since the end of 2019, the virus remained almost unchanged, still in the form that has made it highly contagious and not very lethal: most of the mutations that have occurred (by March 2020 there were more than 160) did not give the virus an evolutionary advantage over humans and were therefore essentially irrelevant.

One year after the outbreak, each country is still struggling to control the pandemic by implementing several different measures. Identifying variants of concern have now become crucial, even though meaningful epidemiological data are still lacking in many cases. A few of the most advanced countries have developed effective epidemiological surveillance systems, while others are still in the process of expanding their current ones. For this reason, data coming from these few countries with an adequate surveillance system tends to influence all the others.

It thus is clear that only when epidemiological surveillance centers will be developed at the national level and then interconnected globally, it will be possible to control the virus in the appropriate ways. This will also allow us to better manage the spreading of variants of concern, and to adopt targeted measures to contain the contagion.

Adaptive mutations in the SARS‐CoV‐2 genome could alter its pathogenic potential, and at the same time would increase the difficulty of drug and vaccine development. This contribution will not deal in detail with the mass of molecular information now available for SARS‐CoV‐2. It will rapidly summarize the information on its evolutionary and structural features that could be useful for the development of vaccines.

Finally, we must remember that identifying these mutations is relevant for the design of antiviral drugs and vaccines updates. For this reason, timely viral detection and sequence analysis, more precise and reliable tracking methods, prompt implementation of measures of social distancing, are fundamental to quickly recognize and contain new emerging clusters of infection.

## CONFLICTS OF INTEREST

All the authors declare that there are no conflicts of interest.

## AUTHOR CONTRIBUTIONS

*Conceptualization*: Davide Zella and Massimo Ciccozzi. *Writing—original draft preparation*: Davide Zella, Marta Giovanetti, Francesca Benedetti, and Massimo Ciccozzi. *Writing—review and editing*: Davide Zella, Marta Giovanetti, Francesca Benedetti, Francesco Unali, Silvia Spoto, Michele Guarino, Silvia Angeletti, and Massimo Ciccozzi. *Supervision*: Massimo Ciccozzi.
